# Resistance to* P. brasiliensis* Experimental Infection of Inbred Mice Is Associated with an Efficient Neutrophil Mobilization and Activation by Mediators of Inflammation

**DOI:** 10.1155/2015/430525

**Published:** 2015-12-24

**Authors:** Felipe Fornias Sperandio, Gisele Pesquero Fernandes, Ana Carolina Silvério Cerqueira Mendes, Giulia Maria de Alencar Castro Bani, Vera Lucia Garcia Calich, Eva Burger

**Affiliations:** ^1^Instituto de Ciências Biomédicas, Universidade Federal de Alfenas (UNIFAL), Brazil; ^2^Setor de Ciências da Saúde, Universidade Federal do Paraná (UFPR), Brazil; ^3^Instituto de Ciências Biomédicas, Universidade de São Paulo (USP), Brazil

## Abstract

Paracoccidioidomycosis (PCM) is a systemic fungal infection, endemic in Brazil, that leads to severe morbidity and even mortality if not correctly treated. Patients may respond differently to PCM depending on the pattern of the acquired immune response developed. The onset of protective immune response is notably mediated by neutrophils (PMN) that play an important role through directly killing the fungi and also by interacting with other cell types to modulate the acquired protective immune response that may follow. In that way, this study aimed to present and compare different experimental models of PCM (intraperitoneal and subcutaneous) regarding PMN production and maturation inside femoral bone marrow and also PMN infiltration in peritoneal and subcutaneous exudates of resistant and susceptible mice. We also assessed the fungal colony forming units and the levels of soluble inflammatory mediators (LTB4, KC, IFN-*γ*, GM-CSF, and IL-10) inside subcutaneous air-pouches to compare the efficiency of the PMN present at this site in relation to the two main neutrophil functions: initial lysis of the invading pathogen and modulation of the acquired immune response.* P. brasiliensis* inoculated intraperitoneally was able to disseminate to the bone marrow of susceptible mice, causing a more marked alteration of PMN production and maturation than that observed after resistant mice infection by the same route. Subcutaneous air-pouch inoculation of* P. brasiliensis* elicited a controlled and limited infection that produced a PMN-rich exudate, thus favoring the study of the interaction between the fungus and the neutrophils. Susceptible mice produced higher numbers of PMN; however, these cells were less effective in killing the fungi. Inflammatory cytokines were more pronounced in resistant mice, which supports their PCM raised resistance.

## 1. Introduction

Paracoccidioidomycosis (PCM) is, out of several other fungal infections, an important and neglected systemic condition that can be easily found in Latin America and especially in Brazil [1]. PCM leads to lung, mucosal, and skin involvement that may comprise acute and even chronic presentation of the disease [2, 3]. Actually, the primary acute PCM infection is later transformed to a chronic phase and its severity depends essentially on the host's immune response [4].

Flaws in immune cell activation and also immune suppression lead to a higher susceptibility to PCM [5, 6]. Accordingly, several inflammatory cells and exceptionally the neutrophils (PMN) are crucial to build the host's response against PCM's fungal agent* Paracoccidioides brasiliensis (Pb)* [7]; such response involves the release and production of antimicrobial factors, cytokines, chemokines, serum antibodies, and so forth [2, 8].

In that way, PMN are mainly constituents of the innate immune response but can also trigger and modulate adaptive chronic reactions when acting upon T cells [9], dendritic cells [10], and B lymphocytes [11]. It is then noteworthy to seek for this inflammatory comportment through experimental models that can mimic the interaction between* Pb *and the immune cells.

Thus, we sought to study different models of* Pb* inoculation in two different mice strains (A/J and B10.A) that are known to be either resistant or susceptible to PCM and that reproduce, respectively, the mild and the severe clinical forms of the disease [12]. Among other differences in behavior, resistant mice (A/Sn and A/J strains) showed low fungal dissemination and caused low mortality, as well as few lesions constituted by active compact and encapsulated granulomas with characteristics indicating effective control of the disease. These mice also showed high activation state of phagocytes, synthesized low levels of specific anti-*Pb* antibodies, with preferential production of IgG2a and IgG3 isotypes, and had preserved delayed type hypersensitivity (DHT) and lymphoproliferative responses and produced preferentially T_H_1 cytokines, constituting the hyperergic pole of experimental paracoccidioidomycosis. On the other hand, susceptible mice (B10.A strain) presented high fungal dissemination, leading to high mortality, numerous loose and disseminated granulomas indicating progression and ineffective control of the disease. Low activation state of phagocytes, high levels of specific antibody, suppressed DTH, and lymphoproliferative responses and a preferential T_H_2 cytokine production pattern were also observed, characterizing the anergic pole of the experimental disease [13].

The presence of PMN, as well as fungal growth or cytokine profile, was assessed and provided a sense of the immunological course regarding bone marrow, peripheral blood, and exudate compositions during* Pb* infection.

## 2. Materials and Methods

### 2.1. Animals

Isogenic male mice, either resistant (A/J) or susceptible (B.10A) to PCM strains [12], approximately 11 weeks old, were kept in controlled temperature rooms under a 12-hour light/12-hour dark cycle and fed with sterile food and distilled water* ad libitum*.

#### 2.1.1. Paracoccidioides brasiliensis

The highly virulent* Paracoccidioides brasiliensis* Pb18 strain [14] was isolated and grown in semisolid Fava Netto media [15]; the fungal culture medium was replaced every 7 days. Pb18 cells were washed thrice with sterile 0.9% saline solution and centrifuged (5810R Centrifuge, Eppendorf, NY, USA) 3x at 1300 g. A fungal suspension containing 10 × 10^6^ yeast cells/mL was measured using a hemocytometer. Fungal cell viability by exclusion of the vital dye Janus Green B [16] was always superior of 75%.

### 2.2. Infection and Inoculation of Mice

B10.A and A/J mice were infected through intraperitoneal routes with a fungal suspension containing 25 × 10^6^/mL and subcutaneously with 0.1 mL of 50 × 10^6^/mL suspension, following a previous inoculation of 2 mL of sterile air, as described by Harmsen and Havell in 1990 [17] and modified by Meloni-Bruneri et al. in 1996 [18]. Control groups were inoculated accordingly with sterile PBS.

### 2.3. PMN Isolation and Quantification

The animals were anesthetized with a lethal dose (0.5 mL of 10% ketamine hydrochloride and 2% Xylazine solution) and PMN were collected from the peritoneum after injection of 3 mL of sterile PBS and further extraction of the whole PBS/exudate solution; 1 mL of this solution was placed on a Suta sedimentation chamber and then the slide was stained with May-Grunwald-Giemsa-Rosenfeld. Differential counting was performed with an optical microscope, in which granulocytes were distinguished from mononuclear cells. Cells were quantified using a hemocytometer and cell viability was assessed with 0.2% Trypan blue (Sigma). Regarding the subcutaneous route, PMN were collected 15 days after the infection of the mice. The animals were anesthetized with a lethal dose (0.5 mL of 10% ketamine hydrochloride and 2% Xylazine solution); cells were collected and placed in sterile tubes with the help of a sterile glass Pasteur pipette after skin flap procedures. The cells were transferred and stored in Falcon tubes containing RPMI (Sigma-Aldrich, St. Louis, MO, USA) with 10% Fetal Bovine Serum (FBS, Sigma), refrigerated (2–6°C), and quantified using a hemocytometer; cell viability was assessed with 0.2% Trypan blue (Sigma).

### 2.4. Identification of PMN Populations

The neutrophils analysis in the bone marrow (myelogram) was done identifying immature neutrophils (promyelocytes, myelocytes, and metamyelocytes) and mature neutrophils (band neutrophils and segmented neutrophils) according to the criteria of Sin and Saintemarie [19]. The cells identification and quantification counts were done collecting material from 6 mice for each model of* P. brasiliensis* inoculation and counting 500 cells from each animal.

### 2.5. Histopathological Evaluation of Intraperitoneal and Air-Pouch Exudates

After euthanasia, biopsies were performed on the infected tissue and the material was fixed in formaldehyde at 10% and followed routine histological procedures to be subsequently embedded in paraffin. Histological sections of 5 *μ*m were obtained and stained with hematoxylin and eosin (HE) or Giemsa.

### 2.6. Quantification of Viable* Pb* through Colony Forming Units

The material was aseptically collected from the subcutaneous air-pouches after 13 days of infection and immediately centrifuged at 1780 g (5810R Centrifuge, Eppendorf, NY, USA). The pellets were resuspended in 100 *μ*L PBS and spread on Petri dishes with the aid of a sterile Drigalski spreader. Similarly, after centrifugation at 1780 g, 100 *μ*L of PMN/*Pb* mixed suspensions obtained after 2 hours of cocultivation was spread on Petri dishes. The experiments were performed in triplicate. The fungal growth on plates was allowed to take place over a period of 12 days, when a paintbrush marker was used to highlight the colonies. The culture medium used in this procedure was BHI agar (HiMedia Laboratories, India) supplemented with 1% glucose, 30% growth factor mixture produced by the fungus itself, and 10% FBS, as described by Singer-Vermes et al. in 1992 [20].

### 2.7. Assessment of Soluble Mediators Concentration inside Air-Pouches

As already described, the material was aseptically collected from the subcutaneous air-pouches after 13 days of infection and centrifuged. The supernatants were collected, filtered using 0.22 *μ*m pore-size filters (Millipore, Bedford, MA, USA), and immediately stored at −70°C. This material was later employed to quantify the concentrations of IFN-*γ*, IL-10, GM-CSF, LB4, and KC using capture enzyme-linked immunosorbent assay (ELISA), following the procedures recommended by the manufacturers' protocols, according to the technique originally described by Coligan et al., 1991 [21]. The antibody pairs used were purchased from Peprotech (Peprotech, Ciudad de Mexico, México) for IFN-*γ*, IL-10, and GM-CSF determination, RD (R&D Systems, Minneapolis, USA) for KC determination, and Cayman (Cayman Chemical, Ann Arbor, Michigan, USA) for LB4 determination. The concentrations of each cytokine were determined with reference to a linear regression curve obtained for the standard curve using twofold dilutions of murine recombinant cytokines. The results are the mean results of three different samples and were expressed as picograms per mL of supernatant collected from air-pouch exsudate.

### 2.8. Statistical Analysis

An ANOVA test was performed, followed by Student's *t*-test to check for differences between experimental groups with a level of significance of 5%. The software used for the analyses was GraphPad Prism 6 (GraphPad Software, Inc.; La Jolla, CA 92037, USA).

## 3. Results

### 3.1. Neutrophilic Population in the Bone Marrow

**Figure 1:**
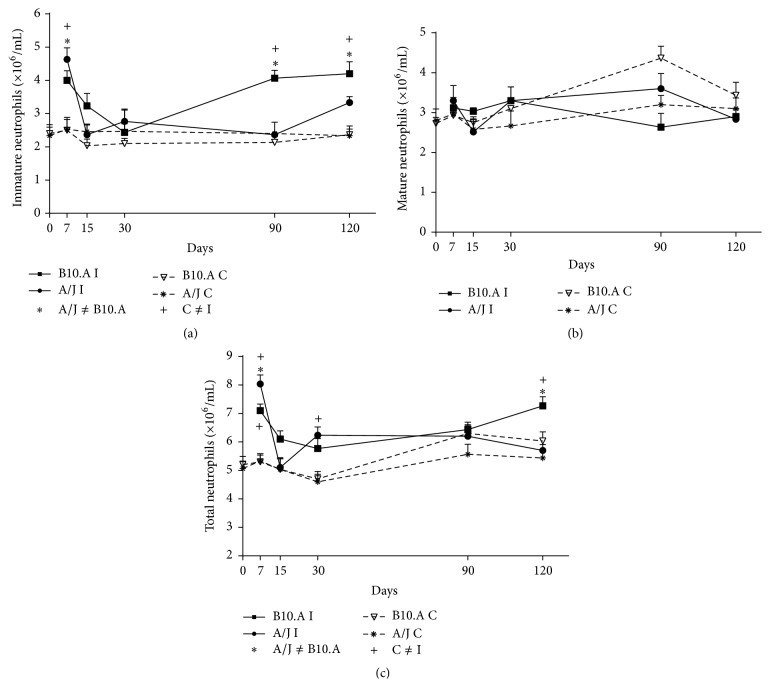
Kinetics of PMN population in bone marrow following intraperitoneal inoculation of* Pb*: (a) immature PMN population after intraperitoneal inoculation; (b) mature PMN population after intraperitoneal inoculation; (c) total PMN population after intraperitoneal route; A/J and B10.A: resistant and susceptible mice, respectively.

Immature PMN were found in higher quantities in the bone marrow of B10.A susceptible than A/J resistant mice after intraperitoneal injection (Figure 1(a)). Concerning mature PMN, we observed higher number of cells in resistant mice after 90 days of intraperitoneal inoculation of* Pb *(Figure 1(b)); no significant differences were found between resistant and susceptible animals in any other period studied (Figure 1(b)). The total number of PMN cells in the bone marrow was significantly higher in susceptible animals after 120 days of intraperitoneal infection (Figure 1(c)).

**Figure 2:**
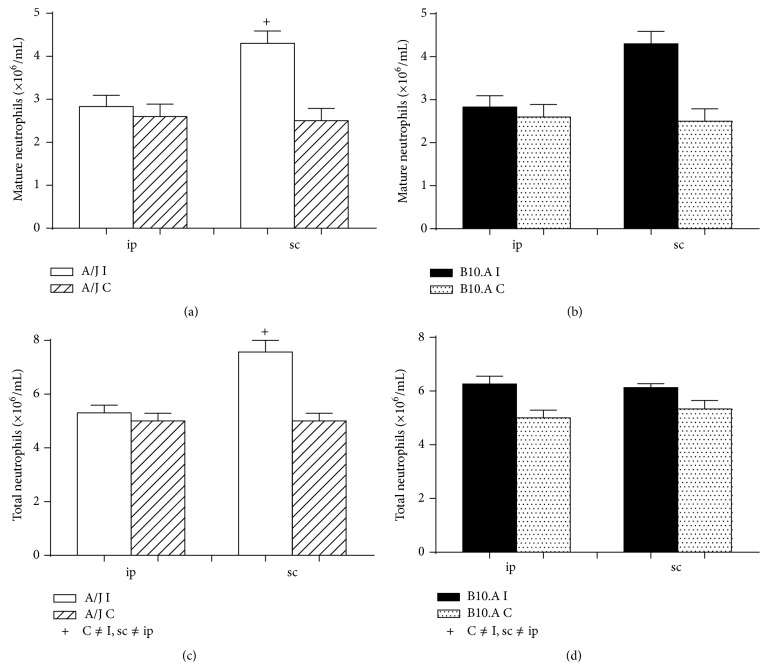
Relative neutrophilic population inside bone marrow between intraperitoneal and subcutaneous routes of* Pb* infection after 15 days: (a) mature cells in A/J resistant mice; (b) mature cells in B10.A susceptible mice; (c) total number of cells in resistant mice; (d) total number of cells in susceptible mice.

Figure 2 shows the relative population of mature and total PMN in the bone marrow of mice that were infected with* Pb *by either an intraperitoneal or subcutaneous route. Statistically significant results are related to the subcutaneous air-pouch infection, in which resistant mice presented higher total and mature neutrophilic population when compared to the intraperitoneal model studied (Figure 2).

### 3.2. Neutrophilic Population in the Peripheral Blood

**Figure 3:**
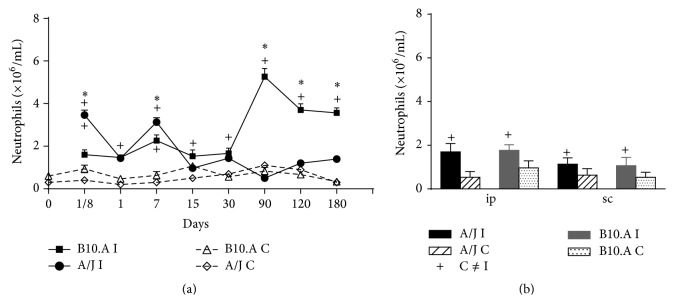
Neutrophilic population in the peripheral blood of resistant A/J or susceptible B.10A mice: (a) kinetics of PMN in mice that had* Pb* inoculated intraperitoneally; (b) relative population of PMN in mice that suffered intraperitoneal and subcutaneous injection of* Pb*.

The kinetic behavior of the total number of PMN inside the blood of resistant and susceptible mice is shown in Figure 3(a) and clearly shows that a larger population of mature neutrophils is present in the blood of susceptible mice that suffered intraperitoneal inoculation.  Figure 3(b) represents the relative population of mature PMN in the blood of susceptible and resistant mice 15 days after contact with* Pb* in either intraperitoneal or subcutaneous environment; there were no statistically significant results.

### 3.3. Neutrophilic Infiltrates in Peritoneum and Subcutaneous Air-Pouch Exudate

**Figure 4:**
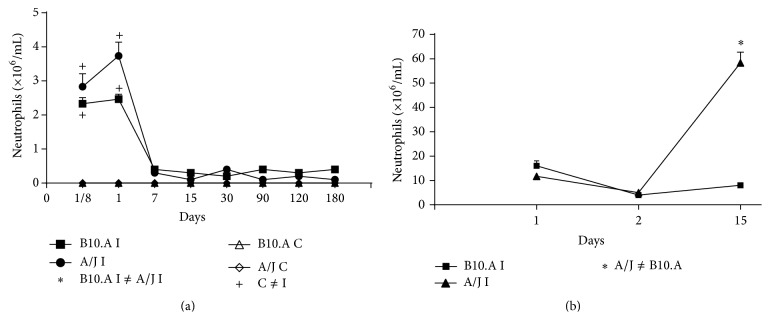
Neutrophilic infiltrates in peritoneum and subcutaneous exudate of A/J resistant and B10.A susceptible mice: (a) kinetics of PMN infiltration in peritoneum; (b) kinetics of PMN infiltration inside subcutaneous air-pouches.

As extracted from Figure 4(a), there is no statistically significant difference in the peritoneal infiltration of PMN of susceptible and resistant mice, and this infiltrate remains low since day 7 and throughout all the subsequent studied periods; however, the air-pouch exudate of air-pouches reveals a prominent mature PMN population at 15 days for the resistant animals (Figure 4(b)).

### 3.4. Histological Appearance of Bone Marrow

**Figure 5:**
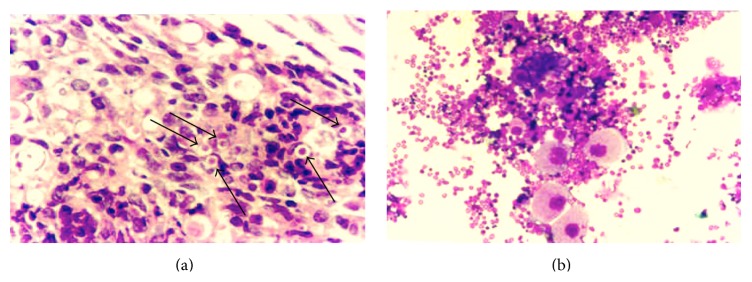
Histological appearance of marrow: (a) conspicuous presence of* Pb* yeasts in susceptible mice 90 days after intraperitoneal infection; (b) evident neutropoiesis along mature megakaryocytes in susceptible mice after 90 days of intraperitoneal infection.

The presence of* Pb* in the femoral marrow of susceptible mice intraperitoneally infected was conspicuous and the morphology of the fungal cells was preserved, suggesting that these cells are viable (Figure 5(a)). Neutropoiesis was also evident along the presence of mature megakaryocytes in the bone marrow of susceptible mice after intraperitoneal infection (Figure 5(b)). As expected, no* Pb* was found inside the marrow of resistant mice [22].

### 3.5. Colony Forming Units of* Pb* and Cytokine Profile in Subcutaneous Air-Pouches

**Figure 6:**
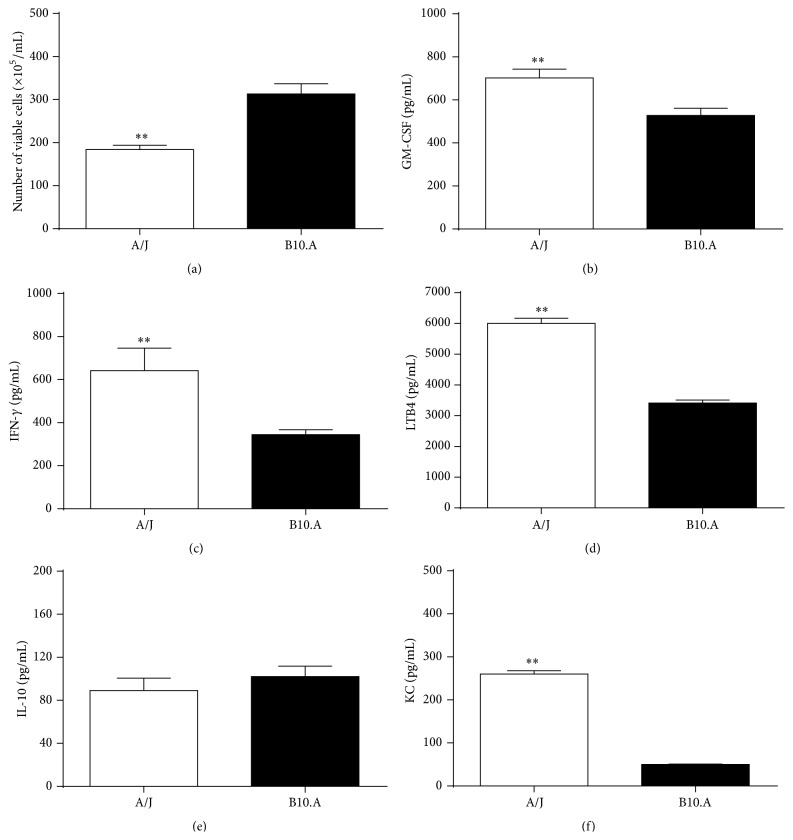
Number of viable* Pb* and concentration of soluble mediators after 13 days of subcutaneous inoculation in A/J resistant and B.10A susceptible mice: (a) colony forming units of* Pb*; levels of (b) GM-CSF, (c) IFN-*γ*, (d) LTB4, (e) IL-10, and (f) KC.

Viable* Pb* were identified inside the subcutaneous air-pouches after 13 days of subcutaneous infection; differences between susceptible and resistant mice favored a great number of fungi inside the subcutaneous air-pouches of susceptible animals (Figure 6(a)). GM-CSF (Figure 6(b)), IFN-*γ* (Figure 6(c)), and LTB4 (Figure 6(d)) levels were significantly augmented in resistant mice as compared to those of susceptible animals. IL-10 levels (Figure 6(e)) were similar between both mouse strains though; and KC concentration inside the subcutaneous air-pouches was shown to be significantly higher in resistant mice (Figure 6(f)).

## 4. Discussion

We included here two routes of* Pb *infection that had already been described in the literature: intraperitoneal [23] and subcutaneous [17, 18]. Three other routes can also be mentioned, intravenous [23], intraoral [24], and intratracheal [25], and were not included in the present investigation. Nevertheless, the subcutaneous air-pouch technique that had already been shown to raise an almost pure pool of PMN, also being a local and controlled* Pb* infection [18, 26], was now compared to other classical methods and did present the highest numbers of mature PMN in marrow, as well as recruited PMN* in loco*. Intraperitoneal injection of* Pb*, on the other hand, leads to an exsudate composed of 40–50% PMN [27].

Interestingly, we found that B10.A susceptible animals produced a more immature population of neutrophils than the A/J resistant mice, after intraperitoneal inoculation of* Pb*. Correspondingly, the intraperitoneal injection of fungi led to a significantly higher production of mature PMN in A/J subjects. We can assume that a PMN population highly composed of mature cells is more effective to fight the fungus and do support the resistant behavior of such mice.

Still, the susceptible animals revealed a higher total number of PMN inside marrow after intraperitoneal fungal injection; this strongly sustains the idea that mature cells (higher percentage in the bone marrow of A/J mice) are more efficient and lead to better resistance once they are highly activated [18, 28], even when in lesser number. At seven days of infection, there were higher quantities of immature PMN in the bone marrow of A/J (resistant mice) than in B10.A (susceptible mice), and at 15 days of infection, there were higher quantities of immature PMN in the bone marrow of B10.A mice than in the bone marrow of A/J mice after intraperitoneal injection (Figure 1(a)).

But at later times of infection, we constantly observed higher quantities of immature PMN in the bone marrow of B10.A mice than in the bone marrow of A/J mice after intraperitoneal injection. This data confirms earlier data observed in this experimental model showing that the susceptible mouse strain is more activated in the beginning of paracoccidioidomycotic infection than the resistant mouse strain. Resistance in this mycosis is due to an efficient delayed type hypersensitivity (DTH) reaction.

In fact, in the later stages of infection, resistant mice show persistent levels of DTH. At the onset of the infection, however, the susceptible mice present a strong DTH reaction, higher than those of the resistant ones, but this reaction is lost throughout the infection, rendering these animals susceptible to paracoccidioidomycosis. A previously published study shows these dynamics of response [29]. Thus, we assume that the higher production of immature PMN by susceptible mice is a tentative to revert their PMN lack of capability. Actually, we were able to show in another study that a competent PMN fungicidal reaction depends more on the level of activation of these cells rather than on their number [26].

In other words, neutrophils of resistant mice gain superior maturation and thus can better deal with a fungal infection. Resistance relies on the activity of immune cells in a way that certain individuals respond better or poorer to PCM depending on their immunological response [2]. Accordingly, it has been classically postulated that PCM patients present deficiencies related to PMN lack of capacity to phagocytose and destroy this fungus [30–33].

On the other hand, it can be argued that one of the consequences of* Pb* infection in susceptible subjects is the impairment of normal neutrophil maturation, with the consequent relative incompetence in dealing with the* Pb* infection. Accordingly, intraperitoneal inoculation of a virulent isolate of* Pb* causes fungal dissemination to various organs. The presence of viable yeast form cells has been detected in the omentum, spleen, liver, and lungs and the numbers were significantly higher in the susceptible mice [22], which is definitely in agreement with our findings, once we could identify morphologically intact* Pb* cells (yeast form) only in the bone marrow of susceptible animals and not in those of the resistant ones. Although we have previously described the dissemination of* P. brasiliensis* to various organs after intraperitoneal infection [22], the presence of the fungus in the bone marrow was not reported until now. Also, in another study [13], we described the histopathology as well as the characteristics of the granulomatous lesions in these organs.

Now, regardless of PMN maturation, the subcutaneous injection of* Pb *was the most efficacious way of obtaining evident neutropoiesis. This subcutaneous infliction was described by Harmsen and Havell in 1990 [17] and modified by Meloni-Bruneri et al. in 1996 [18]. Remarkably, our results show that the subcutaneous route of infection can be utilized to study the inflammatory reaction, particularly of neutrophils, upon* Pb* infection. We cannot affirm that this particular route truly mimics clinical PCM though; but actually, no studied experimental model can represent PCM in its totality.

We saw the highest production of neutrophils (total and mature) with the subcutaneous model. Nevertheless, there is no benefit in inducing a prevalent production of mature cells if such cells cannot reach the site of infection. Intriguingly though, we were able to see that the subcutaneous air-pouch route could also recruit a high number of PMN to the infection site; that means not only a superior production of PMN, but also an evident migration of these cells to the actual subcutaneous pouch. Here, we also showed an obvious presence of mature PMN in peripheral blood for the two experimental methods; the intraperitoneal infection yielded higher differences between resistant and susceptible mice regarding PMN total numbers; higher numbers of PMN were expressive in susceptible animals.

Comparing the marrow results with the peripheral blood findings, we conclude that there was an advanced recruitment of PMN cells in susceptible mice, especially after 90 days of intraperitoneal infection; susceptible animals probably are stimulated to produce higher quantities of immature PMN that rapidly migrate and can be found in the blood. Nevertheless, the intraperitoneal migration of such mature cells is not favored in either of the studied mice models. Moreover, the mature population is much more expressive in the exudate of subcutaneously injected A/J mice, which again proves that neither the higher production inside marrow nor the higher number of blood PMN is responsible for PCM resistance if these cells are not able to reach the site of* Pb* infection.

So, resistant mice apparently recruit PMN to air-pouches in a more “clever” way, inducing higher numbers of mature PMN to remain inside the pouch and thus fight the fungus. That is actually why we can see a neutrophil-rich exudate in subcutaneous air-pouches in outbred Swiss animals that behave similarly to resistant mice [26]. Thus, we favor the use of the subcutaneous air-pouch model of experimental PCM to study inflammatory reaction, especially the acute response, once air-pouches produce a localized infection that can be better managed by the animal itself [34].

Still, the sole participation of inflammatory cytokines and neutrophil recruitment was not sufficient to completely eradicate the* Pb* infection, which was evidenced by the persistent presence of fungi inside the air pockets after 15 days of inoculation [18]. Nevertheless, resistant mice were able to deal better with the infection, which was proved by a significantly lower amount of* Pb* inside their air-pouches. Still, cytokines that effectively activate neutrophils, such as GM-CSF and IFN-*γ* [35, 36], are present in elevated quantities inside the pouches, these quantities being always higher in the resistant mice.

GM-CSF and IFN-*γ* are actually examples of mediators that can increase the fungicidal activity of PMN through a mechanism that relies on the production of ROS such as H_2_O_2_ and superoxide anion [35, 36]; then, our results support a direct match between higher proportion of mature cells and raised participation of GM-CSF inside air-pouches. Accordingly, LTB4, which certainly modulates the fungicidal activity of monocytes and is highly delivered by PMN when in contact with* Pb* [37], is also more existent in A/J animals. LTB4 certainly modulates the fungicidal activity of monocytes and is highly delivered by PMN when in contact with* Pb*; a recent study also shows the role of LTB4 in promoting fungicidal activity of macrophages against* Pb* infection [38].

KC, on the other hand, is chemoattractant for murine PMN [39]; neutrophilic infiltrates of lungs in the early acute phases of* Pb* infection have been related to KC release [39]. Hence, in this study, KC numbers were low for the susceptible group and parallel to a high participation of the anti-inflammatory cytokine IL-10, once after 15 days the inflammatory reaction is already losing its acute identity. However, although IL-10 participation was similar in A/J mice, the KC levels surpassed IL-10 inside the air pockets, again showing a higher recruitment of PMN for this resistant group, even after 15 days of* Pb *inoculation. In accordance, IL-10 negatively modulates the innate response against* Pb*, being related to a Th2 response [37, 40].

Therefore, the cytokine milieus in the air-pouches of resistant mice have an overall inflammatory pattern, able to direct the acquired immune response towards an efficient response to cope with the later phases of PCM, as opposed to the pattern of cytokines present in the exsudate of susceptible mice air-pouches.

## 5. Conclusions

Our results favor the use of subcutaneous air-pouches in order to study the inflammatory reaction produced against the* Pb* infection, once it leads to a higher PMN production and maturation in bone marrow, as well as neutrophilic recruitment to the site of infection, and eases the collection of such immune cells by generating a controlled and localized infection. We also highlight the importance of the host's general immune panorama, in a way that resistant animals present distinct inflammatory responses from that of susceptible ones. This is highlighted by the efficient maturation and migration patterns of neutrophils from the resistant mice, which are present in high concentrations and exposed to inflammatory, activating cytokines at the site of the infection as opposed to what is observed in the susceptible mice. Therefore, neutrophils from resistant mice are able to efficiently cope with the* Pb* at the air-pouch lesion, as well as direct the upcoming acquired immune response towards an efficient, mostly Th1 protective pattern. Again, neutrophils of susceptible mice are less competent to directly deal with the* Pb* present at the site of the infection and modulate the acquired immunity to a mostly regulatory Th2 pattern.
